# Clinically silent early proximal seal failure with suspected interval distal endograft displacement after hybrid zone 2 thoracic endovascular aortic repair in severe arch tortuosity: a case report with 1-year follow-up

**DOI:** 10.3389/fmed.2026.1935630

**Published:** 2026-09-03

**Authors:** Fengsheng Yin, Liang Wang, Lingli Tang, Long Yang

**Affiliations:** 1 Vascular Surgery Department, Chengdu Integrated TCM & Western Medicine Hospital, Chengdu University of Traditional Chinese Medicine, Chengdu, China; 2 Department of Orthopedics, Chengdu Integrated TCM & Western Medicine Hospital, Chengdu University of Traditional Chinese Medicine, Chengdu, China; 3 Teachers College, Chengdu University, Chengdu, China

**Keywords:** aortic arch aneurysm, endograft displacement, hybrid zone 2 repair, proximal seal failure, thoracic endovascular aortic repair, type Ia endoleak

## Abstract

**Background:**

Durable hybrid zone 2 thoracic endovascular aortic repair depends on adequate healthy sealing length, appropriate oversizing, and close endograft apposition. Severe arch tortuosity can compromise these requirements even when completion angiography is satisfactory.

**Case presentation:**

A 46-year-old woman with a primary non-post-surgical distal arch and proximal descending thoracic aneurysm underwent left carotid-subclavian bypass, left subclavian artery origin occlusion, and zone 2 thoracic endovascular aortic repair. The reported proximal landing segment was approximately 16 mm, and the proximal aortic diameter was 24.2 mm. Two 28-mm GORE TAG modules were implanted. Completion angiography showed no visible endoleak. Predischarge CTA on postoperative day 9 demonstrated suspected interval distal displacement of the proximal endograft, loss of the proximal seal, and a high-flow type Ia endoleak. An additional 28-mm proximal extension was deployed immediately distal to the left common carotid artery, restoring the seal. Imaging at 1 month, 4 months, and approximately 12 months showed stable endograft configuration, persistent bypass patency, no recurrent endoleak, and reduced radial thickness of the excluded sac.

**Conclusion:**

A satisfactory completion angiogram did not exclude early proximal seal failure in this severely tortuous arch with a short zone 2 landing segment. Early postoperative CTA enabled treatment before clinical deterioration. The case supports careful anatomical selection, centreline-based reporting, and early surveillance when standard TEVAR is used outside preferred sealing-zone geometry.

## Background

Thoracic endovascular aortic repair is established for descending thoracic aortic disease, but durable exclusion requires an adequate healthy landing zone and stable endograft apposition. These conditions are difficult to achieve in the arch because curvature and pulsatile loading can create incomplete lesser-curvature apposition and reduce effective fixation. Bird-beak configuration is associated with type Ia endoleak and other adverse device-related outcomes after arch TEVAR (1–5).

Hybrid zone 2 TEVAR combines intentional coverage of the left subclavian artery with surgical or endovascular revascularization when needed. This approach extends repair into the segment between the left common carotid and left subclavian arteries, but it does not remove the mechanical requirements for sufficient healthy covered seal (6–9). We report clinically silent early proximal seal failure with interval distal endograft displacement after apparently satisfactory hybrid zone 2 TEVAR. Urgent proximal relining restored the seal, with stability documented at approximately 12 months. This case report was prepared in accordance with the CARE guidelines (Supplementary File 1).

## Case presentation

A 46-year-old woman presented with persistent bilateral headache, neck and shoulder pain, and elevated blood pressure for more than 3 months. Her history included hypertension for more than 3 years and hyperlipidaemia. She had no previous aortic operation, childhood or adult coarctation repair, thoracic trauma, diabetes mellitus, infection, autoimmune disease, or known familial aortic disease. She had no reported phenotypic features of Marfan or Loeys-Dietz syndrome. The lesion was therefore considered a primary, non-post-surgical aneurysm, with a clinically presumed degenerative aetiology. No tissue specimen was available for histopathological confirmation.

At admission, blood pressure was 147/112 mmHg, heart rate was 72 beats/min, temperature was 36.8 °C, and body mass index was 21.6 kg/m^2^. Physical examination showed no major abnormality. Laboratory tests revealed a preoperative serum creatinine level of 66 μmol/L (0.75 mg/dL), corresponding to an estimated glomerular filtration rate of 102 mL/min/1.73 m^2^ using the CKD-EPI equation. A preoperative transthoracic echocardiogram demonstrated normal left ventricular systolic function with an ejection fraction of 63% (modified Simpson’s biplane method).

Transthoracic echocardiography raised clinical suspicion of a tortuous and dilated distal aortic arch. A dedicated multiphase CTA (non-ECG-gated, 1.0-mm slice thickness, comprising unenhanced, arterial, and 70-s delayed phases) subsequently established the definitive lesion anatomy and dimensions.

The multiphase CTA demonstrated severe tortuosity and folding from the distal edge of the LCCA ostium across the LSA region into the proximal descending thoracic aorta (Supplementary Video 1). The aneurysmal segment began in Ishimaru zone 2, involved the adjacent zone 3 segment, and extended into the proximal descending thoracic aorta (Ishimaru zone 4). The maximal preoperative outer aortic dimensions were 57.4 × 56.4 mm on the supplied axial image. The LSA ostium was aneurysmally dilated.

**Figure 1:**
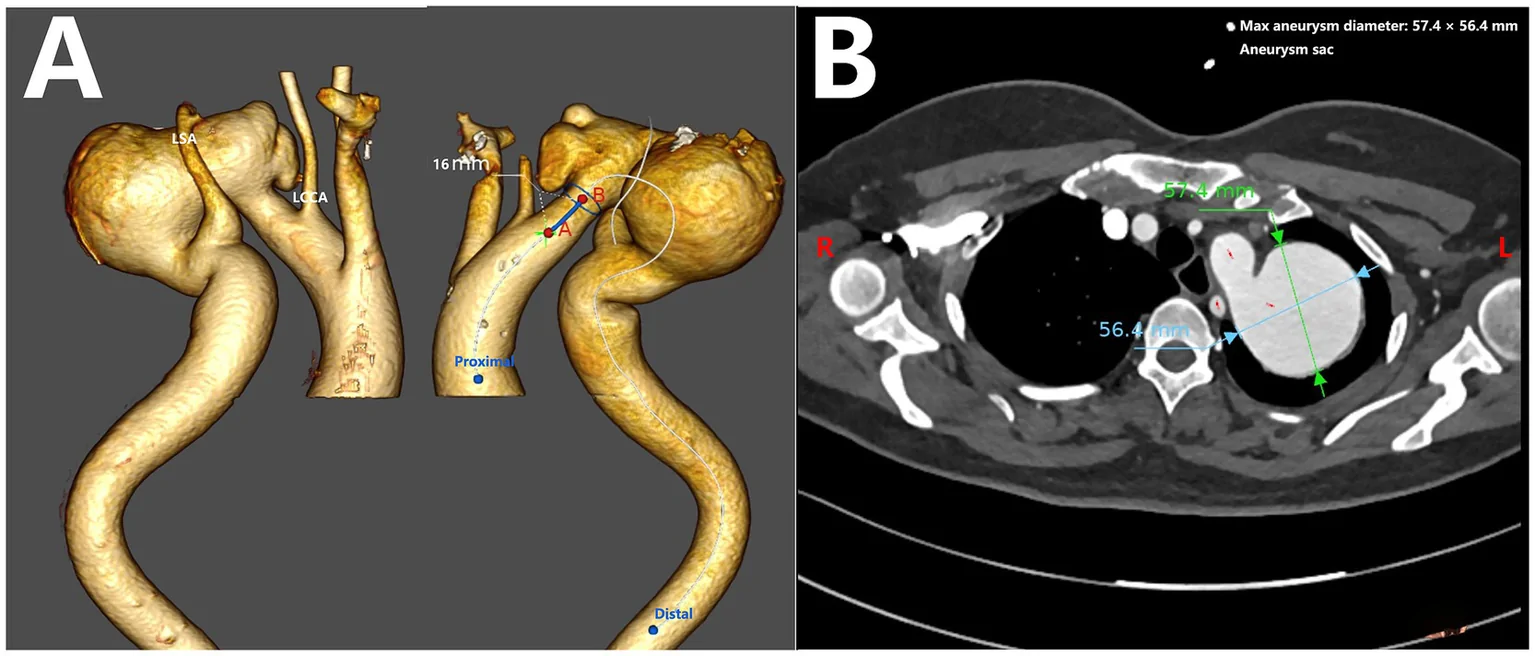
Preoperative CTA showing severe distal arch tortuosity, the reported short proximal landing segment, and aneurysm size. **(A)** Three-dimensional reconstruction and planning view. The reported 16-mm distance extends from the distal edge of the LCCA ostium to the proximal margin of the aneurysmal segment, measured along the aortic centreline. **(B)** Axial CTA showing maximal outer aortic dimensions of 57.4 × 56.4 mm. CTA, computed tomography angiography; LCCA, left common carotid artery; LSA, left subclavian artery.

Centreline planning reported approximately 16 mm from the distal edge of the LCCA ostium to the proximal margin of the aneurysmal segment, measured along the aortic centreline (Figure 1). When evaluated separately along the arch curvatures following initial endograft deployment, the effective covered apposition length (defined as the continuous longitudinal length of graft fabric in direct apposition against healthy aortic wall from the distal LCCA ostium to the proximal aneurysmal margin) was only 12 mm along the inner (lesser) curvature and 20 mm along the outer (greater) curvature, compromised by lesser-curvature malapposition. The proximal aortic diameter, measured strictly perpendicular to the centreline, was 24.2 mm, and the distal landing-zone diameter was 23.5 mm. The aneurysmal segment length was approximately 63 mm. The ascending aorta measured 30.7 × 27.4 mm, and the mid-descending thoracic aorta measured approximately 28 mm.

**Figure 2:**
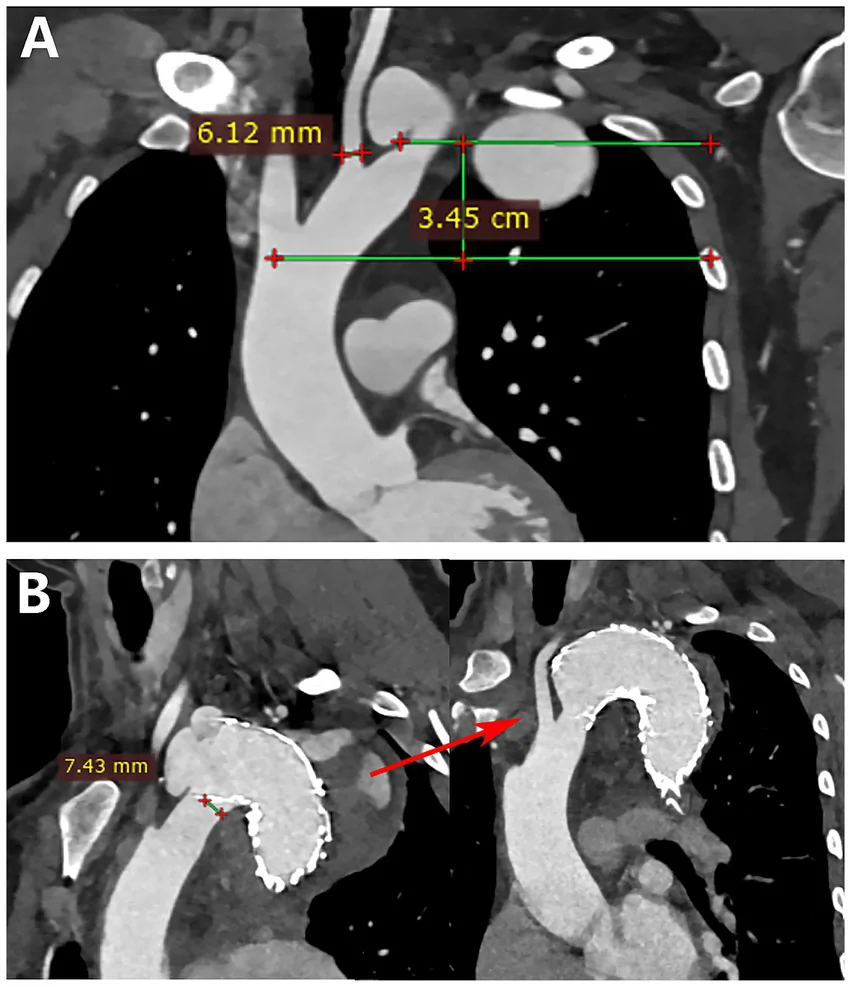
Preoperative aortic arch classification and postoperative visual verification of bird-beak resolution. **(A)** Coronal multiplanar reconstruction demonstrating a Type III aortic arch configuration, with the vertical distance from the origin of the innominate artery to the superior border of the aortic arch measuring 34.5 mm, which markedly exceeds two diameters of the left common carotid artery (LCCA diameter: 6.1 mm). **(B)** Annotated multiplanar reconstruction comparison demonstrating the elimination of the adverse bird-beak configuration following urgent proximal relining, with the malapposed lesser-curvature gap reducing from 7.43 mm prior to extension to 0 mm post-revision, visually confirming complete wall apposition along the inner curvature.

The anatomy was objectively classified as a type III aortic arch based on coronal multiplanar CTA reconstructions, following standard anatomical classification frameworks evaluating arch height relative to reference branch-vessel diameters (10, 11). Specifically, the vertical distance from the origin of the innominate artery to the superior border of the aortic arch measured 34.5 mm, which markedly exceeded two diameters of the left common carotid artery (LCCA diameter: 6.1 mm; 2×6.1mm=12.2mm ; Figure 2A). The arch-to-descending-aorta angle was measured at 18° on centreline-reformatted CTA (using OsiriX MD software on the non-ECG-gated dataset) by fitting intersecting vectors to the distal arch and proximal descending centreline segments.

After multidisciplinary review, open arch replacement was recommended as the conventional durable option for a young patient with this anatomy. The patient declined open cardiothoracic surgery. A single-stage hybrid zone 2 strategy was selected.

## Treatment and follow-up

The index procedure was performed under general anaesthesia. The LCCA was exposed through a left cervical incision, and the mid-LSA was exposed through a left infraclavicular incision. An end-to-side left carotid-subclavian bypass was constructed using a 6-mm expanded polytetrafluoroethylene graft. Angiography confirmed bypass patency, and systemic heparinization was administered.

The left common femoral artery was accessed with a 7F sheath and preclosed with two Perclose ProGlide devices. The left radial artery was accessed with a 5F sheath. Preoperative CTA assessment confirmed adequate access vessel morphology for large-bore sheath navigation: the minimum luminal diameters were 7.35 mm for the left common femoral artery, 7.57 mm for the external iliac artery, and 8.02 mm for the common iliac artery, with mild wall calcification but no significant luminal stenosis or severe tortuosity. The delivery system utilized a 20F GORE DRYSEAL Flex introducer sheath (nominal outer diameter 7.5 mm).

The aneurysmal LSA origin was occluded with an Amplatzer Vascular Plug II, model 9-AVP2-016, with a nominal diameter of 16 mm. To ensure precise anatomical placement and avoid access-site interference, two distinct endovascular routing strategies were utilized for plug deployment and landmark visualization. First, for vascular plug delivery, a catheter and guidewire were navigated from the femoral arterial access into the left subclavian artery (LSA). A long introducer sheath was subsequently exchanged over the wire into the LSA, through which the vascular plug was delivered and successfully deployed at the proximal LSA origin. The LSA diameter at the landing site was 11.7 mm, corresponding to a calculated oversizing of 36.8%. The plug’s final position was 8.7 mm proximal to the left vertebral artery origin and 97.5 mm proximal to the bypass anastomosis, preserving an unobstructed outflow. Second, to establish an angiographic deployment landmark in the ascending aorta without occupying the femoral TEVAR delivery pathway, a 5F pigtail catheter was introduced via the left radial artery access (5F sheath). The catheter was navigated retrogradely through the left brachial and distal subclavian arteries, steered across the newly constructed end-to-side left carotid-subclavian bypass graft into the LCCA, and advanced down through the LCCA ostium into the aortic arch and ascending aorta.

A 0.035-inch Zoe Track stiff guidewire was positioned, and a 20F W. L. Gore introducer sheath was advanced from the femoral access into the descending thoracic aorta. Two 28 × 28 × 150-mm GORE TAG thoracic endoprostheses, model TGU282815, were deployed distal first and then proximal. This distal-first deployment sequence was chosen to establish a stable distal landing column prior to precise proximal positioning, avoiding the passage of a second device through a newly fixed proximal edge in a severely angulated arch. Two identical 28-mm modules were selected because the proximal and distal landing diameters were nearly identical. Nominal oversizing was calculated as [(nominal graft diameter—centreline–perpendicular vessel diameter)/centreline–perpendicular vessel diameter] × 100, corresponding to 15.7% proximal oversizing and 19.1% distal oversizing (based on the 23.5-mm distal landing diameter), with a planned overlap that resulted in an achieved component overlap of 110.6 mm (substantially exceeding the 50-mm manufacturer threshold for same-size components) to maximize composite column stability. Balloon moulding of the sealing zones and component overlaps was deliberately avoided during both the index procedure and the revision due to the severely tortuous arch anatomy and the associated risk of aortic wall injury or retrograde dissection.

**Figure 3:**
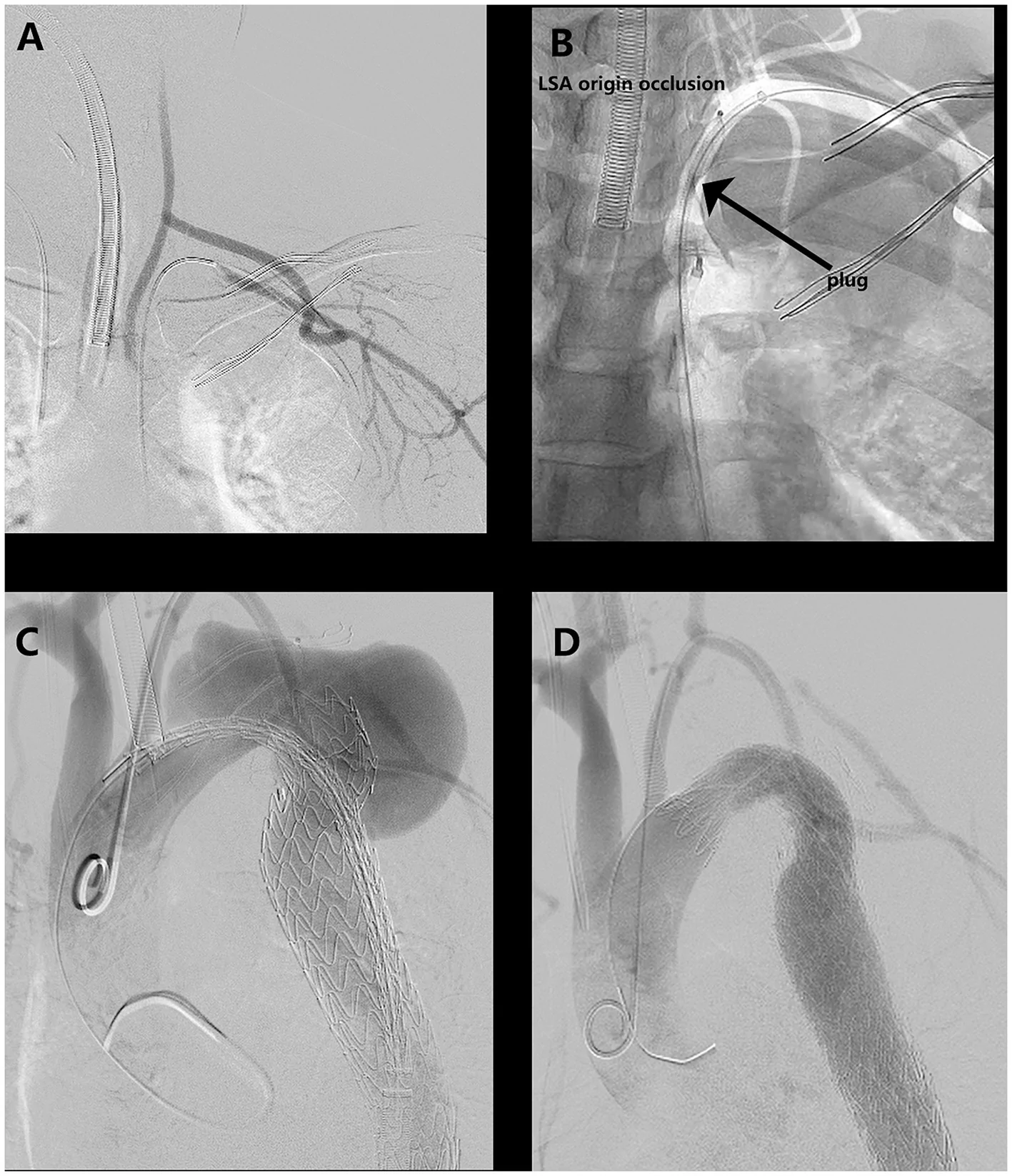
Index hybrid zone 2 TEVAR. **(A)** Predeployment angiography. **(B)** Occlusion of the LSA origin with a 16-mm Amplatzer Vascular Plug II. **(C)** Deployment of two overlapping 28-mm GORE TAG modules with an ascending-aortic pigtail landmark. **(D)** Completion angiography showing no visible endoleak and a patent carotid-subclavian bypass. LSA, left subclavian artery; TEVAR, thoracic endovascular aortic repair.

Manufacturer specifications identify an approximately 4-mm partially uncovered proximal stent segment for the TGU282815 configuration. This segment is not a flared free-flow sealing zone and does not increase covered seal length. The covered proximal edge was aligned as close as feasible to the distal edge of the LCCA ostium. Completion angiography showed aneurysm exclusion without a visible endoleak and preserved bypass flow (Figure 3). The procedural exposure metrics for the index intervention were as follows: total procedure duration was 185 min, fluoroscopy time was 35 min, dose-area product was 125 Gy cm^2^, and total iodinated contrast volume was 135 mL (Iopromide, Ultravist, 370 mgI/mL). The patient required 1 day of postoperative intensive care unit (ICU) monitoring. To maintain prosthetic bypass and endograft patency, postoperative systemic heparinization was bridged to oral rivaroxaban in accordance with standard vascular principles. The immediate postoperative course was uneventful, with no occurrence of stroke, transient ischaemic attack, spinal cord ischaemia, acute kidney injury, access-site complications, bleeding requiring transfusion, cranial nerve injury, wound or lymphatic complications, bypass-related complications, or upper-limb ischaemia.

Predischarge CTA on postoperative day 9 showed distal displacement of the proximal endograft relative to the LCCA landmark, loss of the proximal seal, and a high-flow type Ia endoleak. The supplied reconstruction reported 17 mm of displacement and a 21.3-mm gap relative to the distal edge of the LCCA ostium. Because completion angiography and postoperative CTA were not acquired with an identical standardized projection and no immediate postoperative CTA was available, these measurements support interval displacement but do not independently prove a precisely calibrated 17-mm migration. The bypass remained patent, and no LSA-origin recanalization was seen.

Urgent reintervention was performed under general anaesthesia. The left femoral artery was re-accessed and preclosed with two Perclose ProGlide devices prior to introducing the 20F sheath. Radial access and the pigtail landmark were repeated. A 0.035-inch Amplatz Extra-Stiff wire supported a 20F introducer sheath. During extension deployment, patient immobility and precision were achieved using ventilator-controlled breath-holding (mechanical apnea), while haemodynamic stability was meticulously managed by the anaesthesiologist targeting a stable systolic blood pressure of 90–110 mmHg with continuous intravenous dopamine support (rapid ventricular pacing was avoided). Following the procedure, definitive haemostasis was achieved by securing the preclosed sutures combined with 5 min of manual compression, requiring no secondary closure devices or surgical repair and resulting in an uneventful access-site outcome without bleeding or vascular complications. The procedural exposure metrics for the revision intervention were as follows: total procedure duration was 65 min, fluoroscopy time was 15 min, dose-area product was 45 Gy cm^2^, and total iodinated contrast volume was 70 mL (Iohexol, Omnipaque, 350 mgI/mL).

A 28 × 28 × 100-mm GORE TAG extension, model TGU282810, was placed more proximally, immediately distal to the LCCA, achieving an 80.2-mm overlap into the original proximal module. This extension also has an approximately 4-mm partially uncovered proximal stent segment. The more proximal covered position and increased composite overlap restored seal and column support without changing nominal diameter. Following this revision, by extending coverage proximally to the LCCA ostium and conforming to the longer outer arch geometry, the effective covered apposition length increased to 24 mm along the inner (lesser) curvature and 32 mm along the outer (greater) curvature. Importantly, multiplanar reconstruction confirmed the complete elimination of the adverse bird-beak configuration, with the malapposed gap decreasing from 7.43 mm prior to extension to 0 mm post-revision (Figure 2B). Completion angiography showed no residual type Ia endoleak.

The patient was discharged 3 days after revision. Although contemporary vascular consensus guidelines generally recommend antiplatelet or antithrombotic therapy following TEVAR and supra-aortic prosthetic debranching, routine antithrombotic agents were intentionally withheld at discharge as an individualized institutional decision. Specifically, our multidisciplinary team weighed the patient’s heightened acute hemorrhagic risks—arising from two sequential major procedures requiring open cervical dissection and repeat 20F arterial access within a 9-day interval—against the potential for graft thrombosis. Because the endovascular construct utilized standard unibody tubular stent grafts without small-diameter bridging branches or fenestrations, and given the high-velocity, unobstructed flow through the 6-mm ePTFE carotid-subclavian bypass, we favored a strategy of rigorous clinical and serial CTA surveillance over pharmacological platelet inhibition. To minimize pulsatile aortic loading and mechanical displacement forces on the thoracic endograft, an oral antihypertensive regimen comprising benidipine hydrochloride (8 mg once daily) and arotinolol hydrochloride (5 mg twice daily) was prescribed, maintaining a documented systolic blood pressure target of 120–140 mmHg during long-term surveillance.

**Figure 4:**
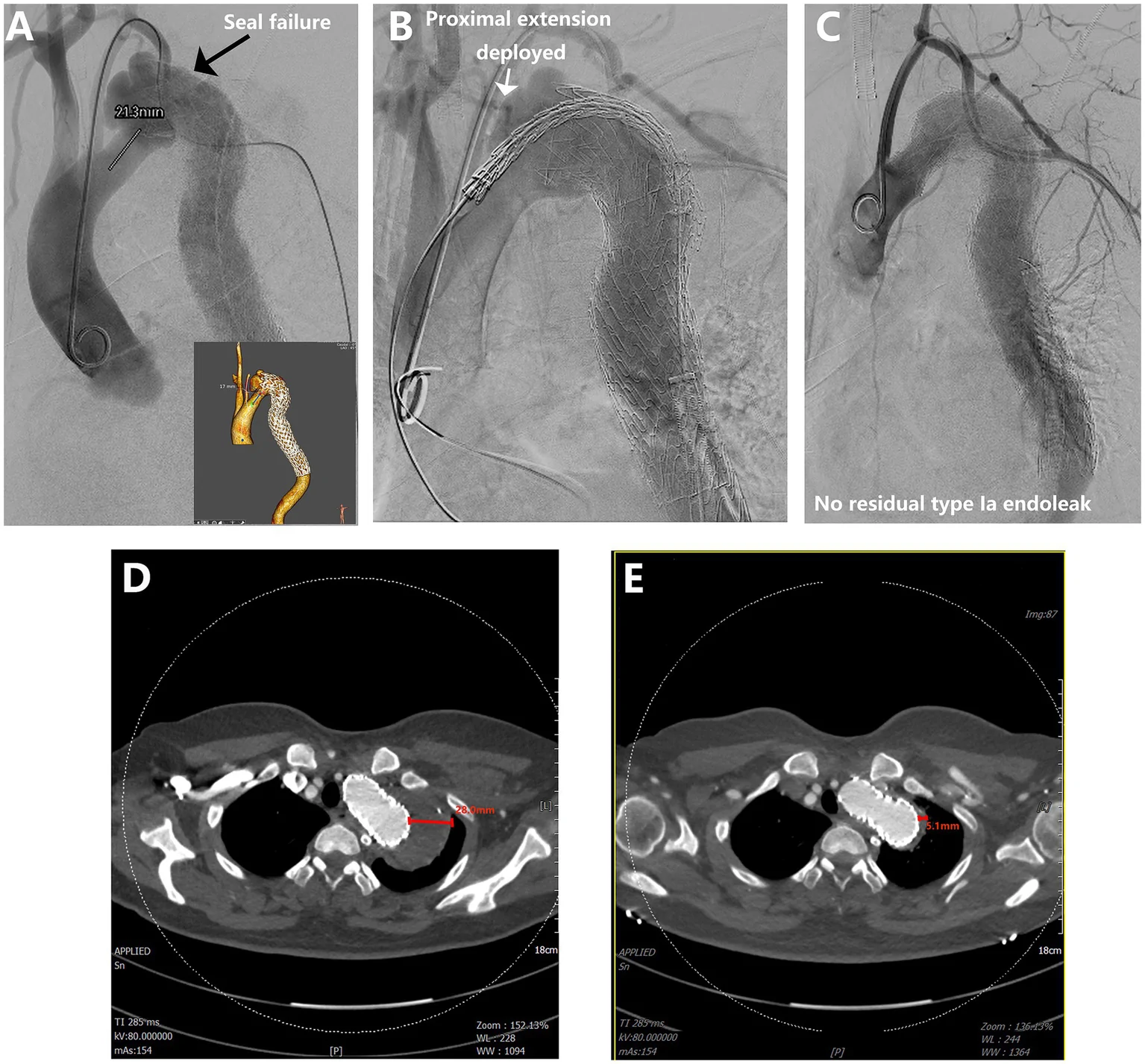
Early proximal seal failure with interval distal endograft displacement and endovascular bailout. **(A)** Postoperative day-9 angiography showing proximal seal failure and a 21.3-mm gap relative to the distal edge of the LCCA ostium. The inset reconstruction reports 17 mm of distal displacement, although identical-projection immediate postoperative imaging was unavailable. **(B)** Proximal extension deployed immediately distal to the LCCA. **(C)** Completion angiography showing no residual type Ia endoleak. **(D,E)** Axial follow-up images at 1 month and approximately 12 months showing reduction in radial excluded-sac thickness from 28.0 mm to 5.1 mm. These values are not maximum sac diameters. CTA, computed tomography angiography; LCCA, left common carotid artery.

Follow-up imaging utilizing a dedicated multiphase CTA protocol (1.0-mm slice thickness, unenhanced, arterial, and delayed phases) at 1 month, 4 months, and approximately 12 months after the index procedure showed stable endograft configuration, no recurrent endoleak, and a patent bypass. The radial thickness of the excluded sac between the graft and outer aortic wall decreased from 28.0 mm at 1 month to 5.1 mm at 12 months on the displayed axial images (Figure 4). These are not maximum sac diameters. Comparable centreline-orthogonal outer-to-outer maximum sac diameters confirmed progressive positive aortic remodelling, measuring 57.4 mm at baseline, 56.2 mm at 1 month, 50.8 mm at 4 months, and decreasing markedly to 43.5 mm at 12-month follow-up (Supplementary Figure 1). No neurologic complication, upper-limb ischaemia, or revision-related adverse event was reported during follow-up.

## Discussion

This case demonstrates clinically silent early proximal seal failure after apparently satisfactory hybrid zone 2 TEVAR in a severely tortuous arch. The key anatomical constraint was the approximately 16-mm reported proximal landing segment. EACTS/STS guidance recommends a healthy proximal sealing zone longer than 25 mm in the distal arch or descending thoracic aorta and 15–20% oversizing for chronic aneurysmal disease (9). The 28-mm graft provided 15.7% nominal oversizing at the reported 24.2-mm proximal diameter, but appropriate diameter selection could not compensate for deficient seal length and severe curvature.

Arch geometry plausibly contributed to failure. Greater arch angulation is associated with bird-beak configuration, and incomplete lesser-curvature apposition is associated with type Ia endoleak (2, 3). A meta-analysis also linked bird-beak configuration with adverse postoperative outcomes after TEVAR (5). These studies support an association among severe angulation, malapposition, and proximal failure. They do not establish that pulsatile force alone caused displacement in this patient.

The imaging supports interval distal displacement because the completion angiogram showed the proximal device adjacent to the LCCA landmark without visible endoleak, whereas day-9 CTA showed a new gap and high-flow type Ia endoleak. Precise migration quantification requires standardized centreline distances from fixed anatomical landmarks and comparable imaging acquisitions (12). The absence of immediate postoperative CTA and the different projection of completion angiography limit certainty. We therefore describe early proximal seal failure with interval distal displacement and retain migration as the most likely, not conclusively proven, mechanism.

Proximal relining addressed the dominant failure mechanism. The extension moved the covered sealing edge closer to the LCCA and increased component overlap and composite column support. The unchanged 28-mm diameter does not mean that the reconstructed geometry was unchanged. A more proximal covered seal and longer supported construct can improve fixation even when nominal radial diameter is identical. Indeed, our anatomical measurements confirmed this mechanical advantage: following relining, the effective covered sealing length increased substantially to 24 mm along the inner curvature and 32 mm along the outer curvature, while achieving an 80.2-mm component overlap and completely eliminating the adverse bird-beak configuration (with the measured gap decreasing from 7.43 mm prior to extension to 0 mm post-revision), thereby securing a durable proximal seal and robust column stability without altering the nominal device diameter.

The 28-mm versus 31-mm sizing decision illustrates a genuine trade-off. The 28-mm device was within the manufacturer’s intended 22-mm to 26-mm aortic range and produced 15.7% oversizing at 24.2 mm. A 31-mm device also covers a 24.2-mm aorta within its intended range and would produce 28.1% oversizing. Greater diameter could increase radial force, but aggressive oversizing in a short, sharply curved arch may worsen lesser-curvature malapposition, bird-beak geometry, or infolding. In retrospect, a 31-mm device might have improved fixation, but the available case evidence cannot establish that it would have prevented seal failure. The broader lesson is that oversizing should not be used to compensate for inadequate healthy seal length.

The TGU282815 and TGU282810 models include an approximately 4-mm partially uncovered proximal stent segment. This design may assist apposition and fixation, but it is not a true flared free-flow segment and cannot provide a fabric seal. Counting it as additional covered landing length would overstate the effective seal in this case.

Open arch replacement remained the conventional durable option for this young patient. For endovascular options, an off-the-shelf standard device combined with a left carotid-subclavian bypass was chosen over a branched or fenestrated endograft after the patient declined open surgery. Relying solely on custom-device manufacturing delay as a rationale is incomplete; device availability, anatomical eligibility, urgency, and operator experience must be evaluated together. At the time of the index procedure, off-the-shelf single-branched devices were not immediately available at our institution for urgent deployment, and importantly, the aneurysmal dilatation involving the LSA ostium rendered the target vessel anatomically unsuitable for a durable branch landing zone. During the urgent revision for high-flow type Ia endoleak, a zone 1 strategy incorporating the LCCA could theoretically have provided a longer proximal seal; however, off-the-shelf zone 1 branched endografts were not locally accessible on an emergency basis, and waiting several weeks for a custom-made device carried an unacceptable risk of sac pressurization and rupture. Given our team’s extensive experience with hybrid arch debranching, urgent proximal relining to seal the endoleak while preserving the patent cervical bypass represented the safest and most effective strategy (8).

Off-the-shelf zone 2 branched platforms represent a significant technological advancement, allowing left subclavian artery (LSA) preservation without cervical debranching in selected patients. Extensive Chinese, European, and emerging Western multicentre experiences with the unibody Castor single-branched endograft have demonstrated favorable midterm patency and low mortality across diverse arch pathologies (13–16). Similarly, initial prospective data for the second-generation Cratos platform show encouraging technical feasibility (17). In parallel, prospective Western trials evaluating the GORE TAG Thoracic Branch Endoprosthesis (TBE) have reported high technical success and durable branch patency in anatomically eligible zone 2 aneurysms (18–20). However, contemporary comparative evidence confirms that these dedicated platforms are not interchangeable; they differ significantly in branch orientation, delivery mechanics, access-vessel requirements, and landing zone specifications (21). In our patient, although iliofemoral access vessels were adequate for large-bore navigation, the documented aneurysmal dilatation of the LSA ostium compromised the branch-specific sealing zone. This anatomical limitation, combined with the lack of immediate emergency institutional availability, reinforces our clinical rationale: anatomical eligibility and urgent device availability—rather than theoretical device capability alone—must dictate the choice between endovascular branch preservation and hybrid arch debranching.

Sex-related considerations are relevant but should not be overstated when evaluating thoracic aortic interventions. Women undergoing TEVAR frequently present with smaller access vessels and more complex arch anatomy, and some registry studies have reported higher perioperative mortality or access intervention rates (22, 23). However, other long-term registry data have not shown a consistent sex-related disadvantage (24). In this patient, preoperative CTA confirmed adequate iliofemoral luminal diameters (7.35–8.02 mm), allowing safe 20F large-bore sheath navigation without access complications. The failure mechanism is therefore better explained by the documented short healthy seal length and severe arch tortuosity rather than sex-specific anatomical limitations.

Early imaging was decisive. The complication was clinically silent and was detected by routine predischarge CTA. Type I endoleak maintains systemic pressure within the excluded sac and requires prompt assessment. Proximal extension eliminated the angiographic endoleak and remained stable through approximately 12 months. The case supports early CTA in selected patients with borderline landing-zone geometry, followed by long-term surveillance because migration-related complications can occur later (1, 12).

This report has limitations. It concerns a single patient and cannot establish comparative effectiveness among open, hybrid, and branched strategies. Immediate postoperative CTA was unavailable, and completion angiography was not obtained with a standardized imaging protocol for precision migration measurement. Longer-term follow-up beyond 12 months is also required to assess ultimate durability.

## Conclusion

Severe arch tortuosity and a proximal landing segment shorter than contemporary preferred sealing-zone recommendations were followed by clinically silent early proximal seal failure with suspected interval distal endograft displacement after hybrid zone 2 TEVAR. Predischarge CTA enabled urgent proximal relining before clinical deterioration. Durable treatment in similar anatomy requires centreline-based planning, device-specific sealing assessment, careful consideration of open or branched alternatives, and early and long-term imaging surveillance.

## Data Availability

The raw data supporting the conclusions of this article will be made available by the authors, without undue reservation.

## Supplementary material

SUPPLEMENTARY FILE 1 Completed CARE checklist for this case report.

SUPPLEMENTARY VIDEO 1 Preoperative rotational three-dimensional CTA demonstrating marked tortuosity and folding of the distal aortic arch and proximal descending thoracic aorta.

SUPPLEMENTARY FIGURE 1 Standardized centerline-orthogonal reconstructions illustrating the progressive regression of the outer-to-outer maximum aneurysm diameter across serial follow-up: 57.4 mm at baseline **(A)**, 56.2 mm at 1 month **(B)**, 50.8 mm at 4 months **(C)**, and decreasing markedly to 43.5 mm at 12 months post-procedure **(D)**.
