# Supplementary material for: Clinically silent early proximal seal failure with suspected interval distal endograft displacement after hybrid zone 2 thoracic endovascular aortic repair in severe arch tortuosity: a case report with 1-year follow-up
Source: Front Med (Lausanne). 2026 Sep 3;13:1935630. doi: 10.3389/fmed.2026.1935630 (PMC13581800; doi:10.3389/fmed.2026.1935630)
Supplement: Supplementary file 1 [file Table_1.docx]

**CARE Checklist for Case Reports**

**Manuscript title:** Clinically silent early proximal seal failure with suspected interval distal endograft displacement after hybrid zone 2 thoracic endovascular aortic repair in severe arch tortuosity: a case report with 1-year follow-up

**Article type:** Case Report

Checklist version: Final submission version

Note: Manuscript locations are reported by section heading rather than page number.

| **CARE item** | **Reporting item** | **Manuscript location** | **Status / details based on the current manuscript** |
| --- | --- | --- | --- |
| **1. Title** | Identify the report as a case report and include the main diagnosis or intervention in the title. | Title | Reported. The title includes the main intervention/diagnostic issue and ends with "a case report". |
| **2. Keywords** | Provide keywords that identify the clinical condition, intervention, and case-report nature where appropriate. | Abstract: Keywords | Reported. The keywords identify thoracic endovascular aortic repair, aortic arch aneurysm, proximal seal failure, endograft displacement, type Ia endoleak, and hybrid zone 2 repair. The case-report nature is explicit in the article type and title. |
| **3. Abstract** | Provide a concise structured summary, including background/novelty, case presentation, and main conclusion. | Abstract | Reported. The abstract is structured with Background, Case presentation, and Conclusions and summarizes the clinically silent early migration in borderline anatomy, urgent bailout extension, and durable 1-year follow-up. |
| **4. Background** | Briefly summarize the clinical context and explain why the case is educational or noteworthy. | Background | Reported. The manuscript explains the difficulty of achieving durable proximal seal in tortuous arch anatomy and introduces the educational value of early postoperative surveillance in off-label/borderline anatomies. |
| **5a. Patient information** | Report de-identified demographic and relevant clinical information. | Case Presentation | Reported. The patient is described as a 46-year-old woman with hypertension. Diabetes mellitus, infection, trauma, and autoimmune diseases are denied. It is explicitly noted she had no familial history of aortic diseases and lacked phenotypical features of connective tissue disorders. |
| **5b. Chief concerns and symptoms** | Describe the patient's chief complaints and presenting symptoms. | Case Presentation | Reported. The manuscript reports persistent bilateral headache and neck/shoulder pain with elevated blood pressure for more than 3 months. |
| **5c. Relevant history** | Include relevant medical, family, psychosocial, genetic, and past-intervention history when available and relevant. | Case Presentation | Reported as available. Relevant medical history and key negative histories are provided. No additional family, psychosocial, or genetic history is reported in the current manuscript. |
| **6. Clinical findings** | Describe relevant physical examination and clinical findings. | Case Presentation | Reported. Admission blood pressure, heart rate, temperature, and physical examination findings are provided. |
| **7. Timeline** | Present important dates and time points from presentation, diagnosis, treatment, reintervention, and follow-up. | Case Presentation; Treatment and follow-up | Reported narratively. The manuscript describes symptoms for more than 3 months, preoperative imaging, index hybrid zone 2 TEVAR, routine predischarge CTA on postoperative day 9, urgent proximal extension, discharge 3 days after revision, and follow-up at 1 month, 4 months, and approximately 12 months. |
| **8a. Diagnostic assessment** | Describe diagnostic tests, imaging, and key findings. | Case Presentation; Treatment and follow-up; Figure legends | Reported. Echocardiography, contrast-enhanced CT/CTA, three-dimensional reconstruction, multiplanar planning, angiography, and follow-up CT/CTA findings are described, including standardized anatomical arch classification and longitudinal diameter tracking. |
| **8b. Diagnostic challenges and reasoning** | Describe diagnostic challenges, differential considerations, and reasoning where relevant. | Case Presentation; Treatment and follow-up; Discussion | Reported. The manuscript explains the diagnostic challenge of detecting clinically silent endograft migration and discusses the biomechanical reasoning (continuous pulsatile displacement forces) causing early failure in a type III arch with a critically short landing zone. |
| **8c. Prognostic or risk-related features** | Report relevant prognostic characteristics or risk-related features when applicable. | Case Presentation; Discussion | Reported. The manuscript identifies severe distal arch tortuosity, folding, a critically short proximal sealing segment, proximal type I endoleak, and subsequent aneurysmal sac regression as relevant risk and outcome features. |
| **9a. Therapeutic intervention** | Describe the type of intervention and key procedural details. | Treatment and follow-up | Reported. The index treatment included left carotid-subclavian bypass, LSA origin occlusion, and hybrid zone 2 TEVAR; bailout treatment consisted of proximal stent-graft extension. |
| **9b. Intervention administration** | Describe how the intervention was performed, including devices, access routes, technical details, and dosing/timing where relevant. | Treatment and follow-up | Reported. Access routes, bypass graft, plug, catheter and sheath details, guidewires, stent-graft models/sizes, deployment sequence, overlap, and angiographic endpoints are described. |
| **9c. Changes in intervention** | Describe changes to the treatment plan and the rationale for those changes. | Treatment and follow-up; Discussion | Reported. Urgent reintervention was performed after postoperative day 9 CTA showed distal migration with a secondary type Ia endoleak; proximal extension was selected because the dominant mechanism was proximal seal loss caused by migration. |
| **10a. Follow-up and outcomes** | Report clinical and imaging outcomes after intervention. | Treatment and follow-up; Discussion | Reported. Follow-up imaging showed stable endograft configuration without recurrent endoleak, maintained bypass patency, and progressive involution of the excluded aneurysmal sac. |
| **10b. Follow-up tests** | Report important follow-up test results. | Treatment and follow-up; Figure legends; Additional files | Reported. Follow-up CTA/contrast-enhanced CT at 1 month, 4 months, and approximately 12 months is reported, including reduction in radial excluded-sac thickness from 28.0 mm to 5.1 mm, and regression of the centerline-orthogonal outer-to-outer maximum aneurysm diameter from 57.4 mm to 43.5 mm. |
| **10c. Adverse events** | Report adverse or unanticipated events and complications. | Treatment and follow-up | Reported. The manuscript states that no neurologic complications, upper-limb ischemia, or revision-related adverse events were observed during follow-up. |
| **11. Discussion** | Discuss strengths, limitations, relevant literature, rationale for conclusions, and key lessons. | Discussion; Conclusion | Reported. The manuscript discusses early failure despite satisfactory completion angiography, the biomechanical vulnerability of standard stent grafts in severely tortuous anatomy, mechanism-directed bailout, the critical need for early postoperative CTA surveillance, and limitations. |
| **12. Patient perspective** | Include the patient's perspective or experience when appropriate, or explain if unavailable. | Not included as a separate manuscript section | Not applicable/not available. A separate patient-perspective statement is not included in the current manuscript because the patient's narrative perspective was not obtained; written consent for publication was obtained. |
| **13. Informed consent** | State whether written informed consent was obtained. | Declarations: Consent for publication | Reported. Written informed consent for publication of clinical details, images, and associated video material was obtained from the patient. |

*Completed CARE checklist for submission as Additional file 1.*
